# Correlates of whole-blood polyunsaturated fatty acids among young children with moderate acute malnutrition

**DOI:** 10.1186/s12937-017-0264-3

**Published:** 2017-07-13

**Authors:** C. W. Yaméogo, B. Cichon, C. Fabiansen, M. J. H. Rytter, D. Faurholt-Jepsen, K. D. Stark, A. Briend, S. Shepherd, A. S. Traoré, V. B. Christensen, K. F. Michaelsen, H. Friis, L. Lauritzen

**Affiliations:** 1 Centre de Recherche en Sciences Biologiques, Alimentaires et Nutritionnelles, Université Ouaga I Prof Joseph KI-ZERBO, Ouagadougou, Burkina Faso; 2 Department of Nutrition, Exercise and Sports, University of Copenhagen, Frederiksberg C, Denmark; 3 Département Biomédical et Santé Publique, Institut de Recherche en Sciences de la Santé, 03 BP 7192 Ouagadougou 03, Burkina Faso; 4 Department of Infectious Diseases, Rigshospitalet, Copenhagen, Denmark; 5 Department of Kinesiology, University of Waterloo, Waterloo, ON Canada; 6 Tampere Centre for Child Health Research, 33014 University of Tampere, Tampere, Finland; 7 Alliance for International Medical Action, Dakar, Sénégal; 8 Médecins Sans Frontières — Denmark, Copenhagen, Denmark

**Keywords:** Essential fatty acids, Undernourished children, Diet, Morbidity

## Abstract

**Background:**

Severe acute malnutrition (SAM) has been associated with low polyunsaturated fatty acid (PUFA) status. However, investigations regarding PUFA status and correlates in children with moderate acute malnutrition (MAM) from low-income countries are scarce. The aim of this study was to describe whole-blood PUFA levels in children with moderate acute malnutrition (MAM) and to identify correlates of PUFAs.

**Methods:**

We conducted a cross-sectional study using baseline data from a prospective nutritional intervention trial among 1609 children with MAM aged 6–23 months in Burkina Faso,West Africa. Whole-blood PUFAs were measured by gas chromatography and expressed as percent of total whole-blood fatty acids (FA%). Potential correlates of PUFAs including infection, inflammation, hemoglobin, anthropometry (difference between children diagnosed as having MAM based on low mid-upper-arm-circumference (MUAC) only, low MUAC and weight-for-height z-score (WHZ), or low WHZ only) and diet were assessed by linear regression adjusted for age and sex.

**Results:**

Children with MAM had low concentrations of whole-blood PUFAs, particularly n-3 PUFAs. Moreover, children diagnosed with MAM based only on low MUAC had 0.32 (95% confidence interval (CI), 0.14; 0.50) and 0.40 (95% CI, 0.16; 0.63) FA% lower arachidonic acid (AA) than those recruited based on both low WHZ as well as low MUAC and those recruited with low WHZ only, respectively. Infection and inflammation were associated with low levels of all long-chain (LC)-PUFAs, while hemoglobin was positively associated with whole-blood LC-PUFAs.

**Conclusion:**

While PUFA deficiency was not a general problem, overall whole-blood PUFA concentrations, especially of n-3 PUFAs, were low. Infection, inflammation, hemoglobin, anthropometry and diet were correlates of PUFAs concentrations in children with MAM.

**Trial registration:**

The trial is registered at http://www.isrctn.com (ISRCTN42569496).

## Introduction

Approximately, 33 million children worldwide suffer from moderate wasting [1]. The current case definition of moderate acute malnutrition (MAM) includes children with moderate wasting, defined as a weight-for-height z-score (WHZ) between -2 and -3, based on the 2006 WHO growth standard [2], and/or children with a mid-upper-arm circumference (MUAC) between 115 and 125 mm [3]. Children with MAM are at immediate risk of morbidity and mortality from infectious diseases [4, 5], and may present with lipid metabolism disturbances likely to impair blood levels of polyunsaturated fatty acids (PUFAs) [6].

Tissue concentrations of PUFAs are reflected in the fatty acid composition of plasma and erythrocytes and the amount of various PUFAs in these pools has been shown to be low in children with severe malnutrition [7, 8]. Limited data exist on early life intake and blood levels of PUFAs in low-income countries among malnourished children [9]. The fatty acid composition in plasma or red blood cells from children with moderate malnutrition has only been investigated in two studies with small samples sizes [7, 10]. However, in these studies included children with moderate stunting and/or underweight rather than wasting and used different growth references rather than the current WHO growth standards from 2006 [2]. No study was found that investigated fatty acids in children with MAM based on the above definition and compared the fatty acid composition in whole-blood between children diagnosed with MAM based on low MUAC only, low MUAC and WHZ, or low WHZ only.

PUFAs play an important role in early life because they are needed to ensure optimal growth and development of all organ systems [11]. More specifically, linoleic acid (LA), α-linolenic acid (ALA), docosahexaenoic acid (DHA) and arachidonic acid (AA) are involved in numerous metabolic processes in addition to acting as a source of energy [12]. LA, incorporated in skin ceramides, is of great importance for skin barrier integrity [13]. DHA is abundant in the cells of the central nervous system and is important for brain development [14]. Furthermore, DHA and AA are incorporated into cell membrane phospholipids and are precursors of lipid mediator signalling molecules [15, 16], which play important roles, among others, in the immune system [17]. Considering these important functions, more attention should be paid to PUFA status in children with MAM and it should be investigated how blood PUFA composition correlates with factors such as diet, infections (e.g. diarrhea, malaria), inflammation and anemia and whether there is an association between PUFAs and anthropometry.

Previous studies investigating blood PUFA composition in Burkina Faso, West Africa, have been undertaken in populations not suffering from MAM and in the central region [18–20], an area of the country where groundnuts are widely available and diets are high in fat [21]. Our study was focused on whole-blood PUFA composition among 6–23 months-old children with MAM from the northern region in Burkina Faso, where food insecurity is common. The aim of the study was 1) to describe whole-blood PUFA composition in children with MAM and 2) to identify correlates (e.g. hemoglobin, acute phase proteins, clinical infection, anthropometry and diet) of PUFA composition.

## Subjects and methods

### Study setting and participants

This is a cross-sectional study using baseline data from a prospective nutritional intervention trial among 1609 children with MAM aged 6–23 months. The study took place in the Province du Passoré, Burkina Faso, at five local governmental health centers (Gomponsom, Latoden, Bagaré, Bokin and Samba). The recruitment area covered a total of 143 villages and a population of approximately 258,000. Recruitment took place from September 2013 to August 2014.

Children, aged 6–23 months, residing in the recruitment area were invited to participate if they were diagnosed with MAM; based on a WHZ between -3 and -2 based on the 2006 WHO growth standard [2] and/or MUAC between 115 mm and 125 mm [3]. Children were excluded if they were treated for SAM, if they were already in a nutritional program or if they had been hospitalized within the past 2 months, had medical complications requiring hospitalization, or a severe disability.

### Socio-demographic, clinical and diet data collection

A local nurse carried out a clinical examination and collected data on sociodemographic characteristics as well as 2 week retrospective morbidity. Fever was defined as an axillary temperature ≥37.5 °C [22]. The nurse also collected data on breastfeeding and the childrens’ diet using structured questionnaires administered to the caretakers by trained interviewers. With regard to breastfeeding, caretakers were asked whether the child was ever breastfed, currently breastfed and if so how many times the child was breastfed in the previous 24 h. Dietary data was collected using a qualitative 24-h recall interview. More specifically, caretakers were asked what the children consumed in the previous 24-h, but no quantities were recorded. If a particular dish was mentioned, caretaker’s were asked to list the ingredients. Information recorded during the qualitative 24-h recall interview was then used to complete the food group list section of the questionnaire by answering “yes” or “no” for each food group. The list of 25 food groups was based on internationally available questionnaires from WHO [23], FAO [24] as well as research about diets in Ouagadougou carried out by the Institut de Recherche et Développement [25] and adapted to the local context using dietary information collected during the Treatfood pilot study. For reasons of simplicity, the 25 food groups was then aggregated into eight food groups, namely i) cereals, roots, and tubers; ii) legumes and nuts; iii) liquid oils and fats; iv) dairy; v) flesh foods; vi) eggs; vii) vitamin A-rich fruits and vegetables or viii) other fruits and vegetables as suggested by WHO [23].

### Blood sampling and analyses

During the medical examination up to 2.5 mL of none fasting venous blood were collected by phlebotomy using needle and syringe. One drop of blood was used to carry out rapid antigen test for *Plasmodium falciparum* malaria (SD Bioline, Malaria antigen P.f.), and a second drop for determination of hemoglobin level by Hemocue (HB 301, Ängelholm, Sweden). A third drop was used to saturate 1 cm^2^ of a chromatography paper strip treated with 50 μg 2,6-di-tert-butyl-4-methylphenol (butylated hydroxytoluene) and 1000 μg deferoxamine mesylate salt (both from Sigma-Aldrich, St. Louis, MI, USA) as described previously [26]. The blood spots were allowed to air dry and then the paper strip were kept in cold boxes in the field and stored in zip lock plastic bags at 4 °C until shipment to Canada. The remaining blood sample was put into a tube with clot activator (BD tube reference #368492; Becton, Dickinson and Company, Franklin Lakes, USA) and transported in a coldbox at 2–8 °C to the trial lab, where serum was isolated following centrifugation at 3000 RPM for 5 min (EBA 20 S Hettich, Tuttlingen, Germany).

Serum was stored at -20 °C until shipment to a laboratory in Germany (Vitamin lab, Willstaett, Germany) for analysis. Serum C-reactive protein (CRP) and α_1_-acid glycoprotein (AGP) were determined using a simple sandwich enzyme-linked immunosorbant assay [27]. The thresholds used for defining abnormal values for CRP and AGP were as follows: CRP >10 mg/L [28] and AGP >1 g/L [29].

The fatty acid composition of whole-blood was analysed as previously described [30, 31]. In brief, fatty acid methyl esters were prepared from dried blood spots by direct trans-esterification in 14% boron trifluoride in methanol (Pierce Chemicals, Rockford, IL, USA) with hexane using a convectional block heater set at 95 °C for 60 min. The organic layer containing the fatty acid methyl esters was collected for analysis on a Varian 3900 gas chromatograph with a CP-8400 autosampler (Varian Inc., Mississauga, ON, Canada) and equipped with a DB-FFAP 15 m × 0.10 mm i.d. × 0.10 μm film thickness, nitroterephthalic acid modified, polyethylene glycol, capillary column (J&W Scientific from Agilent Technologies, Mississauga, ON, Canada). Fast gas chromatography settings with hydrogen as the carrier gas were used as previously described [30, 31]. Peaks were identified by retention times through comparison to an external mixed standard sample (GLC-462, Nu Chek Prep Inc., Elysian, MN, USA). Whole-blood fatty acid concentration is given as (μg fatty acid/100 μL whole-blood) and fatty acid composition data are given as weight percent of individual fatty acids relative to the total fatty acid concentration in each sample (FA%).

Due to lack of cut-off points for whole-blood Mead acid and n-6 docosapentaenoic acid (DPA), the ratios Mead acid:AA, n-6 DPA:DHA or n-6/n-3 PUFAs were used to define PUFA deficiency. Based on data in healthy well-nourished Danish infants (Lauritzen et al. unpublished data), we considered a Mead acid:AA ratio > 0.02 as an indicator of PUFA deficiency and a ratio of n-6 DPA:DHA > 0.2 and a n-6/n-3 PUFA ratio > 10.5 was taken to indicate low n-3 PUFA status.

### Anthropometric measures

Anthropometric measurements were done by trained staff, after standardisation sessions. Weight was measured in duplicate to the nearest 100 g with an electronic scale (Seca model 881 1021659, Seca GmbH & Co. KG, Hamburg, Germany) with double weighing function. Length was measured in duplicate to the nearest 1 mm with a wooden length board. MUAC was measured to the nearest 1 mm at the midpoint between the olecranon and the acromion process using a standard measuring tape.

### Data analysis

The data were doubled entered into Epidata 3.1 software (Epidata Association, Odense, Denmark). The statistical analysis and tests were performed in Stata 12 (StataCorp, College Station, TX, USA). A WHO WHZ table was used during recruitment at sites, but for data analysis WHZ scores were recalculated using the STATA package “zscore06”. Variables were tested for normality using Shapiro-Wilk tests and histograms. Results are shown as mean ± standard deviation (±SD) or median (interquartile range (IQR)). The difference between groups was analyzed by one-way analysis of variance (ANOVA) and p-values from post-hoc pairwise comparisons (three comparisons for each ANOVA test) were Bonferroni adjusted. Multivariable linear regression analysis was performed to assess correlates of individual PUFAs in children with MAM adjusted for age and sex. Statistical significance was set at *p* < 0.05.

## Results

Among the 1609 recruited children, a total of 1572 (97.7%) children had whole-blood fatty acids determined and 37 (2.3%) did not either due to failure to draw blood or problems during blood analysis at the lab. Upon recruitment, 457 (29%), 786 (50%), and 329 (21%) were diagnosed with MAM based on “MUAC only”, “MUAC and WHZ”, and “WHZ only”, respectively, as previously reported [32]. The median (IQR) age was 11.3 (8.2; 16) months, half (54.8%) were girls (Table 1) and 95% were still breastfed. As reported elsewhere [33], co-morbidity was common. Among the 1572 children with fatty acid data, 593 (38.1%) children had been ill in the 2 weeks before admission based on maternal recall – hereof 318 (20.2%) with fever and 277 (17.6%) with diarrhea. At recruitment, 629 (40.2%) had a positive malaria test, 275 (17.5%) had fever and 83 (5.3%) had diarrhea. The mean hemoglobin was 10.0 (±1.6) g/dL, while the median CRP and AGP were 2.3 (0.8; 9.5) mg/L and 1.2 (0.9; 1.6) g/L, respectively, and 364 (23.6%) had elevated CRP and AGP.

**Table 1:** Background data, anthropometric, clinical and biochemical characteristics among 1572 children with moderate acute malnutrition

Age (months), median (IQR)	11.3 (8.2; 16.0)
6-11, n (%)	854 (54.3)
12-17, n (%)	447 (28.5)
18-23, n (%)	271 (17.2)
Sex	
Boys, n (%)	682 (45.2)
Girls, n (%)	710 (54.8)
Breastfeeding status	
Still breastfed, n (%)	1488 (94.8)
Not breastfed, n (%)	82 (5.2)
Anthropometrics indicators, mean (±SD)	
WHZ	−2.2 (±0.5)
HAZ	−1.7 (±1.1)
MUAC (mm)	122.6 (±4)
Biochemical	
Hemoglobin (g/dL), mean (±SD)	10.0 (±1.6)
AGP (g/L), median (IQR)	1.2 (0.9; 1.6)
CRP (mg/L), median (IQR)	2.3 (0.8; 9.5)
Morbidity	
Ill the last two weeks	
No, n (%)	967 (61.9)
Yes, n (%)	596 (38.1)
Diarrhea at recruitment	
No, n (%)	1489 (94.7)
Yes, n (%)	83 (5.3)
Fever at recruitment	
No, n (%)	1295 (82.5)
Yes, n (%)	275 (17.5)
Malaria at recruitment	
No, n (%)	936 (59.8)
Yes, n (%)	629 (40.2)

Data are total numbers (%), mean (±SD) or median (interquartile range (IQR): 25^th^; 75^th^ percentile) as indicated. Numbers in categories may not sum up 1572 due to missing data, e.g. data on breastfeeding were available from 1570, on AGP or CRP from 1541, on ill the last two weeks from 1563, on fever from 1570 and on malaria from 1565

The majority (85.2%) of the children had consumed cereals, roots and tubers in the previous 24 h, while 285 (18.1%), 237 (15.1%), and 454 (28.9%) had consumed liquid oils and fats, legumes and nuts or vitamin A-rich fruits and vegetables, respectively (Table 4). More than half (54.5%) of the children had also consumed others fruits and vegetables. Almost all children were breastfed (95%) and only 6 (0.4%), 60 (3.8) and 107 (6.8%) had eaten eggs, dairy and flesh foods, respectively, on the day before the examination.

### Concentrations and correlates of polyunsaturated fatty acids

The mean weight percent of whole-blood LA and ALA were 16.22 (±2.26) and 0.21 (±0.09) FA%, respectively, while that of their respective LC-PUFAs, AA and DHA, were 7.08 (±1.53) and 1.64 (±0.53) FA%, respectively (Table 2). The mean Mead acid:AA ratio of 0.01 (±0.00) and the generally low mean weight percent of Mead acid (0.07 (±0.03) FA%) indicates that essential fatty acid deficiency was not frequent in the participants. Based on our definition, only 1.21% of the children with MAM had signs of PUFA deficiency. However, the mean ratio of 0.25 (±0.10) between n-6 DPA and DHA and the mean n-6/n-3 PUFA ratio of 11.23 (±2.85) indicates low n-3 PUFA status. The percents of children who were above the mean ratio of 0.25 (±0.10) between n-6 DPA and DHA or 11.23 (±2.85) between n-6 and n-3 PUFA were 51.2 and 45.8% respectively.

**Table 2:** Whole blood fatty acids composition in 1572 children with moderate acute malnutrition

		Mean (±SD)
Saturated fatty acid		45.29 (±2.99)
Monounsaturated fatty acid		22.57 (±3.39)
Polyunsaturated fatty acid (PUFA)		28.52 (±2.78)
n-6 PUFA		25.99 (±2.65)
Linoleic acid (LA)	18:2n-6	16.22 (±2.26)
Dihomo-y-linolenic acid	20:3n-6	0.85 (±0.21)
Arachidonic acid (AA)	20:4n-6	7.08 (±1.53)
Adrenic Acid	22:4n-6	0.95 (±0.26)
n-6 Docosapentaenoic acid (DPA)	22:5n-6	0.38 (±0.12)
n-3 PUFA		2.48 (±0.65)
α-Linolenic acid (ALA)	18:3n-3	0.21 (±0.09)
Eicosapentaenoic acid (EPA)^a^	20:5n-3	0.13 (0.10; 0.18)
n-3 Docosapentaenoic acid	22:5n-3	0.43 (±0.12)
Docosahexaenoic acid (DHA)	22:6n-3	1.64 (±0.53)
Mead acid	20:3n-9	0.07 (±0.03)
Mead acid:AA	20:3n-9/20:4n-6	0.01 (±0.00)
n-6 DPA:DHA	22:5n-6/22:6n-3	0.25 (±0.10)
n-6 PUFA:n-3 PUFA		11.13 (±2.85)

Data are given as mean (± standard deviation, SD) in weight percent relative to total fatty (FA%), if not otherwise indicated. Mean total fatty acid concentration was 417 (±183) μg/100 μL whole blood in study children. ^a^Median (interquartile range: 25^th^; 75^th^ percentile)

Comparisons using ANOVA showed that children recruited based on MUAC only had lower AA than those recruited based on both MUAC and WHZ (6.88 vs. 7.15 FA%, *p* = 0.007) and WHZ only (6.88 vs. 7.19 FA%, *p* = 0.013), but higher ALA than those recruited based on WHZ (0.22 vs. 0.20 FA%, *p* = 0.042) (Table 3). Furthermore, children recruited based on low MUAC only had lower EPA than those recruited with WHZ only (0.12 vs. 0.14 FA%, *p* = 0.040). Furthermore, multiple regression analyses, adjusted for age and sex, revealed that the children who were recruited based on MUAC only had 0.32 (95% confidence interval (CI), 0.14; 0.50) FA% lower AA than those recruited based on both MUAC and WHZ and 0.40 (95% CI, 0.16; 0.63) FA% lower AA than those recruited based on WHZ only. Recruitment based on MUAC only was also associated with 0.09 (95% CI, 0.01; 0.17) FA% lower DHA than those recruited based on WHZ only, whereas recruitment based on both MUAC and WHZ was associated with a higher ratio of n-6 DPA relative to DHA (0.01 [95% CI, 0.00; 0.02]) and higher Mead acid (0.004 [95% CI, 0.000; 0.007] FA%) compared to those recruited with WHZ only. Being recruited based on MUAC only compared to WHZ only was positively associated with Mead acid:AA ratio (β = 0.001, 95% CI, 0.000; 0.002). Although median age differed between children recruited based on MUAC only, both MUAC and WHZ, or WHZ only, the difference in AA, DHA and Mead acid:AA across recruitment criteria was not confounded by age, sex and breastfeeding.

**Table 3:** Whole blood fatty acids composition by recruitment criteria in 1572 children with moderate acute malnutrition

		MUAC (*n*= 457)	MUAC and WHZ (*n*= 786)	WHZ (*n*= 329)
		Median age (IQR) 9.6 (7.3; 13.6)	Median age (IQR) 11.6 (8.4; 16.4)	Median age (IQR) 13.3 (9.9; 17.8)
		Mean FA (±SD)	Mean FA (±SD)	Mean FA (±SD)
Saturated fatty acid		45.35 (±3.38)	45.29 (±2.82)	45.24 (±2.82)
Monounsaturated fatty acid		22.77 (±3.53)	22.57 (±3.34)	22.29 (±3.31)
Polyunsaturated fatty acid (PUFA)		28.40 (±2.69)	28.54 (±2.78)	28.68 (±2.92)
n-6 PUFA		25.91 (±2.53)	25.99 (±2.67)	26.11 (±2.77)
Linoleic acid (LA)	18:2n-6	16.37 (±2.16)	16.15 (±2.31)	16.22 (±2.29)
Dihomo-y-linolenic acid	20:3n-6	0.87 (±0.21)	0.85 (±0.21)	0.85 (±0.21)
Arachidonic acid (AA)	20:4n-6	6.88 (±1.53)^1,2^	7.15 (±1.53)	7.19 (±1.54)
Adrenic Acid	22:4n-6	0.92 (±0.26)^2^	0.96 (±0.27)	0.97 (±0.27)
n-6 Docosapentaenoic acid (DPA)	22:5n-6	0.37 (±0.13)	0.39 (±0.13)	0.38 (±0.13)
n-3 PUFA		2.42 (±0.64)	2.50 (±0.68)	2.51 (±0.62)
α-Linolenic acid (ALA)	18:3n-3	0.22 (±0.09)^2^	0.22 (±0.09)	0.20 (±0.09)
Eicosapentaenoic acid (EPA)^a^	20:5n-3	0.12 (0.09; 0.18)^2^	0.13 (0.10; 0.18)	0.14 (0.10; 0.20)
n-3 Docosapentaenoic acid	22:5n-3	0.43 (±0.12)	0..45 (±0.13)	0.44 (±0.12)
Docosahexaenoic acid (DHA)	22:6n-3	1.60 (±0.52)	1.65 (±0.54)	1.67 (±0.53)
Mead acid	20:3n-9	0.07 (±0.03)	0.07 (±0.03)	0.07 (±0.02)
Mead acid:AA	20:3n-9/20:4n-6	0.01 (±0.01)	0.01 (±0.00)	0.01 (±0.01)
n-6 DPA:DHA	22:5n-6/22:6n-3	0.25 (*±*0.10)	0.25 (*±*0.10)	0.24 (*±*0.09)
n-6 PUFA:n-3 PUFA		11.40 (2.91)	11.10 (2.86)	11.00 (2.77)

Data are given as mean (±standard deviation, SD) weight percent of total whole blood fatty acids, if not otherwise indicated. p-value for difference between admission groups are based on ANOVA test with Bonferroni adjustment. *Abbreviations*: *WHZ* weigh-for-height z-score, *MUAC* mid-upper arm circumference

^1^ *p* <0.05 between MUAC and, MUAC and WHZ, and ^2^ *p* <0.05 between MUAC and WHZ

^a^Median (interquartile range: 25^th^; 75^th^ percentile) and ANOVA test on log value

Diet was found to be a correlate of whole-blood PUFA status adjusted for age and sex (Table 4). Relative to children who were not breastfed, breastfed children had 1.17 (95% CI, 0.64; 1.70), 0.75 (95% CI, 0.39; 1.11), and 0.13 (95% CI, 0.01; 0.25) FA% higher LA, AA, and DHA, respectively. Children who had consumed foods from the legumes and nuts group or the oils and fats group in the previous 24 h had 0.38 (95% CI, 0.16; 0.59) and 0.32 (95% CI, 0.12; 0.52) FA% lower AA, respectively, compared to those who did not consume these foods. This was also the case for children with a dietary intake from the vitamin A-rich fruits and vegetables group. Children with a dietary intake from the oils and fats group or the flesh food group had 0.08 (95% CI, 0.09; 0.15) and 0.23 (95% CI, 0.12; 0.33) FA% higher DHA, respectively compared to those who had not eaten these foods. Dietary intake from the flesh foods group was found to be associated with a higher proportion of DHA relative to that of AA and that was also the case for dietary intake of oils, which were mainly vegetable oils. Dietary intake from both of these food groups was also found to provide a larger difference in the proportion of n-3 PUFAs than in n-6 PUFAs.

**Table 4:** Correlations between individual whole blood polyunsaturated fatty acid and dietary intake of various food componentes based on a 24-hour recall in 1572 children with moderate acute malnutrition

	n (%)	n-6 polyunsaturated fatty acids			n-3 polyunsaturated fatty acids			Ratios	
		LA 18:2n-6	AA 20:4n-6	n-6 DPA 22:5n-6	ALA 18:3n-3	DHA 22:6n-3	Mead acid 20:3n-9	Mead acid/AA 20:3n-9/20:4n-6	n-6 DPA/DHA 22:5n-6/22:6n-3
Grains, roots, and tubers group									
No	231 (14.8)	Ref	Ref	Ref	Ref	Ref	Ref	Ref	Ref
Yes	1335 (85.2)	−0.05 (-0.38 ; 0.27)	0.01 (-0.21 ; 0.22)	0.006 (-0.01 ; 0.02)	−0.01 (-0.02; 0.01)	0.03 (-0.04 ; 0.10)	0.001 (-0.003; 0.004)	0.000 (-0.001; 0.001)	−0.00 (-0.01; 0.01)
Legumes and nuts group									
No	1335 (84.9)	Ref	Ref	Ref	Ref	Ref	Ref	Ref	Ref
Yes	237 (15.1)	0.07 (-0.24 ; 0.38)	−0.38 (-0.59 ; -0.16) *	−0.03 (-0.04 ; -0.01) **	0.02 (0.00; 0.03)*	−0.05 (-0.13 ; 0.01)	−0.000 (-0.004; 0.003)	0.001 (0.000; 0.001)*	−0.01 (-0.02; 0.01)
Oils and fats group									
No	1286 (81.9)	Ref	Ref	Ref	Ref	Ref	Ref	Ref	Ref
Yes	285 (18.1)	−0.01 (-0.31 ; -0.28)	−0.32 (-0.52 ; -0.12)**	−0.04 (-0.05 ; -0.02)**	−0.02 (-0.01; 0.01)	0.08 (0.09 ; 0.15)*	0.001 (-0.002; 0.004)	0.001 (0.000; 0.002)**	−0.03 (-0.04; -0.01)**
Dairy group									
No	1512 (96.2)	Ref	Ref	Ref	Ref	Ref	Ref	Ref	Ref
Yes	60 (3.8)	−0.41 (-0.99 ; 0.17)	0.002 (-0.39 ; 0.39)	0.02 (-0.01 ; 0.05)	0.01 (-0.02; 0.03)	0.01 (-0.12 ; 0.15)	0.008 (0.002; 0.010)*	0.001 (-0.000; 0.020)	0.01 (-0.01; 0.04)
Flesh foods group									
No	1464 (93.2)	Ref	Ref	Ref	Ref	Ref	Ref	Ref	Ref
Yes	107 (6.8)	−0.24 (-0.69 ; 0.20)	−0.18 (-0.49 ; 0.11)	−0.03 (-0.06 ; -0.01)*	−0.01 (-0.03; 0.00)	0.23 (0.12 ; 0.33)**	−0.003 (-0.008; 0.002)	−0.000 (-0.001; 0.006)	−0.04 (-0.05; -0.02)*
Eggs group									
No	1561 (99.6)	Ref	Ref	Ref	Ref	Ref	Ref	Ref	Ref
Yes	6 (0.4)	−1.11 (-2.93; 0.70)	−0.96 (-2.19; 0.26)	−0.02 (-0.12; 0.08)	0.04 (-0.03; 0.12)	−0.04 (-0.47; 0.37)	−0.004 (-0.020; 0.010)	0.001 (-0.003; 0.005)	−0.01 (-0.08; 0.07)
Vitamin A-rich fruits and vegetables group									
No	1116 (71.1)	Ref	Ref	Ref	Ref	Ref	Ref	Ref	Ref
Yes	454 (28.9)	0.15 (-0.1; 0.40)	−0.24 (-0.41; -0.07)*	−0.02 (-0.03; -0.00)**	0.01 (-0.00; 0.02)	0.01 (-0.05; 0.07)	−0.000 (-0.003; 0.002)	0.000 (-0.000; 0.001)	−0.01 (-0.02; 0.00)
Others fruits and vegetables group									
No	715 (45.5)	Ref	Ref	Ref	Ref	Ref	Ref	Ref	Ref
Yes	857 (54.5)	−0.06 (-0.29; 0.17)	0.10 (-0.05; 0.26)	0.12 (-0.00; 0.02)	−0.01 (-0.02; -0.00)*	0.03 (-0.02; 0.09)	0.001 (-0.001; 0.004)	0.000 (-0.000; 0.001)	−0.00 (-0.01; 0.01)
Breastfeeding									
No	82 (5.2)	Ref	Ref	Ref		Ref	Ref	Ref	Ref
Yes	1488 (94.8)	1.17 (0.64; 1.70)**	0.75 (0.39; 1.11)**	0.00 (-0.02; 0.03)	−0.01 (-0.03; 0.01)	0.13 (0.01; 0.25)*	−0.020 (-0.030;-0.020)**	−0.005 (-0.006;-0.004)**	−0.04 (-0.06; -0.01)**

Data are given as regression coefficients (95% confidence interval) in weight percent of total whole blood fatty acid concentration after adjustment for age and sex. *Abbreviations*: *LA* Linoleic acid, *AA* Arachidonic acid, *n-6DPA* n-6 Docosapentaenoic acid, *ALA* α-Linolenic acid, *DHA* Docosahexaenoic acid, *ref* reference group. Numbers in categories may not sum up to 1572 due to missing data. Data on intake of grains, roots, and tubers group were available for 1566 children, on intake of flesh foods from 1571, eggs from 1567, on vitamin A-rich fruits and vegetables and breastfeeding from 1570. *p<0.05; **p<0.01

Morbidity and biochemical markers of infection were also correlates of whole-blood PUFA content after adjustment for age and sex (Table 5). Children who had fever at recruitment had lower LA, AA and DHA by 0.52 (95% CI, 0.23; 0.82), 0.29 (95% CI, 0.09; 0.49) and 0.10 (95% CI, 0.03; 0.17) FA%, respectively. Similarly, fever in the 2 weeks prior to recruitment was associated with low AA and DHA, but not LA. Diarrhea at recruitment was negatively associated with LA, but positively associated with DHA, which was also seen with diarrhea based on maternal recall (*p* < 0.05, data not shown). Children with elevated CRP had 0.91 (95% CI, 0.74; 1.10) and 0.23 (95% CI, 0.17; 0.30) FA% lower AA and DHA, respectively, but 0.04 (95% CI, 0.02; 0.05) FA% higher ALA. Elevated AGP was also associated with lower AA and DHA (0.60 [95% CI, 0.43; 0.76] and 0.10 [95% CI, 0.04; 0.15] FA%, respectively) but higher ALA (0.02 [95% CI, 0.01; 0.03] FA%). A positive malaria test was associated with lower AA and DHA (1.06 [95% CI, 0.91; 1.20] and 0.30 [95% CI, 0.25; 0.35] FA%, respectively) and higher ALA (0.05 [95% CI, 0.04; 0.05] FA%). Overall, elevated serum CRP and AGP, and a positive malaria test were associated with a higher Mead acid:AA ratio. A positive malaria test was also associated with a higher n-6 DPA/DHA ratio. For each 1 g/dl increase in hemoglobin, LA and ALA decreased by -0.22 (95% CI, -0.29; -0.15) and -0.02 (95% CI, -0.02; -0.01) FA%, respectively. However, for each 1 g/dl increase in hemoglobin, AA and DHA increased by 0.39 (95% CI, 0.35; 0.44) and 0.11 (95% CI, 0.10; 0.13) FA%, respectively.

**Table 5:** Biochemical and morbidity correlates of individual polyunsaturated fatty acid in 1572 children with moderate acute malnutrition

	n (%)	n-6 polyunsaturated fatty acids			n-3 polyunsaturated fatty acids			Ratios	
		LA 18:2n-6	AA 20:4n-6	n-6 DPA 22:5n-6	ALA 18:3n-3	DHA 22:6n-3	Mead acid 20:3n-9	Mead acid/AA 20:3n-9/20:4n-6	n-6 DPA/DHA 22:5n-6/22:6n-3
Hemoglobin									
g/dl	1572 (100)	−0.22 (-0.29; -0.15)**	0.39 (0.35; 0.44)**	0.02 (0.01 ; 0.02)**	−0.02 (-0.02; -0.01)**	0.11 (0.10; 0.13)**	0.000 (-0.001; 0.001)	−0.001 (-0.001; -0.001)**	−0.004 (-0.008;-0.001)**
≥11	472 (30.0)	Ref	Ref	Ref	Ref	Ref	Ref	Ref	Ref
<11	1100 (70.0)	0.73 (0.49; 0.98)**	−0.77 (-0.93; -0.61)**	−0.03 (-0.05; -0.02)**	0.03 (0.02; 0.04)**	−0.27 (-0.33; -0.21)**	−0.001 (-0.004; 0.002)	0.001 (0.007; 0.002)**	0.02 (0.01; 0.03)**
CRP									
<10mg/L	1166 (75.7)	Ref	Ref	Ref	Ref	Ref	Ref	Ref	Ref
≥10mg/L	375 (24.3)	−0.04 (-0.30 ; 0.23)	−0.91 (-1.10; -0.74)**	−0.05 (-0.06 ; -0.03)**	0.04 (0.02; 0.05)**	−0.23 (-0.30; -0.17)**	−0.005 (-0.008; -0.002)**	0.001 (0.000; 0.001)**	0.01 (-0.00; 0.16)
AGP									
<1g/L	516 (33.5)	Ref	Ref	Ref	Ref	Ref	Ref	Ref	Ref
≥1g/L	1025 (66.5)	0.01 (-0.22; 0.25)	−0.60 (-0.76; -0.43)**	−0.02 (-0.04 ; -0.12)**	0.02 (0.01; 0.03)**	−0.10 (-0.15; -0.04)*	0.001 (-0.001; 0.004)	0.001 (0.001; 0.002)**	0.00 (-0.01; 0.10)
Diarrhea									
No	1489 (94.7)	Ref	Ref	Ref	Ref	Ref	Ref	Ref	Ref
Yes	83 (5.3)	−0.64 (-1.14; -0.14)*	0.20 (-0.13; 0.55)	0.02 (-0.01; 0.05)	−0.02 (-0.04; 0.00)	0.16 (0.05; 0.28)**	−0.004 (-0.010; 0.001)	−0.001 (-0.002; 0.000)	−0.02 (-0.04; 0.00)
Fever									
No	1295 (82.5)	Ref	Ref	Ref	Ref	Ref	Ref	Ref	Ref
Yes	275 (17.5)	−0.52 (-0.82; -0.23)**	−0.29 (-0.49; -0.09)**	−0.01 (-0.02; 0.005)	0.01 (-0.00; 0.02)	−0.10 (-0.17; -0.03)**	−0.005 (-0.009; -0.002)**	−0.000 (-0.001; 0.000)	0.01 (-0.00; 0.02)
Malaria									
No	936 (59.8)	Ref	Ref	Ref	Ref	Ref	Ref	Ref	Ref
Yes	629 (40.2)	0.20 (-0.02; 0.43)	−1.06 (-1.20; -0.91)**	−0.02 (-0.04; -0.01)**	0.05 (0.04; 0.05)*	−0.30 (-0.35; -0.25)**	0.002 (-0.001; 0.004)	0.002 (0.001; 0.003)**	0.02 (0.01; 0.03)**

Data are given as regression coefficients (95% confidence interval) in weight percent of total whole blood fatty acids concentration after adjustment for age and sex. *Abbreviations*: *LA* Linoleic acid, *AA* Arachidonic acid, *n-6DPA* n-6 Docosapentaenoic acid, *ALA* α-Linolenic acid, *DHA* Docosahexaenoic acid, *ref* reference group. Numbers in categories may not sum up to 1572 due to missing data, e.g. data on CRP and AGP were available for 1541 children, on fever from 1570, on malaria from 1565. *p<0.05; **p<0.01

## Discussion

While we found that PUFA deficiency was not a general problem in children with MAM, overall PUFA concentrations, especially of n-3 PUFAs, were low. There could be a number of explanations for this. Firstly, the low levels of whole-blood n-3 PUFAs found in children with MAM could be linked to their diet [34]. The children were from a population with a mainly plant-based diet rich in LA, including peanut oils and peanut based-products, cottons seed oils and cereals. Infants and young children in such a population will have an n-3 PUFA intake far below the recommended intake [35]. Previous studies from the central region in Burkina Faso have demonstrated a high proportion of n-6 PUFAs relative to n-3 PUFAs in breast milk of healthy mothers nursing 5-months-old infants and this was negatively associated with growth [18]. The low relative levels of n-3 PUFAs in children with MAM was compensated for by a high proportion in SFA, which is in line with data from moderately wasted Pakistani children [6]. This high proportion of SFA in our study could be due to more breastfeeding in Burkina Faso as shown in previous studies [18, 36]. Secondly, the low whole-blood n-3 PUFAs could be due to metabolic disturbances occuring with malnutrition [6]. Micronutrient deficiencies such as zinc and iron combined with protein deficiency may impair delta-6 desaturase activity [5, 37] and decrease the conversion of ALA to DHA.

As expected, breastfeeding, flesh foods, vegetable oils and fats were found to be correlates of PUFAs in children with MAM. Breast milk is recognised as a good source of LC-PUFAs [38], although this is influenced by maternal diet [39]. Children who were breastfed on the day before blood sampling had higher LA, AA, and DHA compared to those who were not breastfed. Children who consumed flesh foods (meat and fish) in the previous 24 h also had higher whole-blood n-3 PUFAs and DHA than those who did not consume these types of foods. This is in line with meat and specifically fish being the dominant sources of n-3 LC-PUFAs [34]. A high intake of n-3 LC-PUFAs has been shown to increase EPA and DHA in plasma and tissue phospholipids [40], which is often compensated by a reduction in LA, AA and n-6 DPA.

With regard to anthropometry, children recruited by MUAC only seem to have lower whole-blood n-6 PUFAs and n-3 PUFAs compared to those recruited by WHZ only. Specifically, recruitment by MUAC only was associated with low AA and DHA and this could be related to a low muscle mass. Low MUAC only has been shown to be related to low arm muscle mass [41], which is generally associated with hypercortisolemia occuring with malnutrition [42]. Hypercortisolemia increases resting energy expenditure fueled by increased oxidation of fat [43], and this may decrease whole-blood LC-PUFA status in children with low MUAC. Another explanation could be that intake of LC-PUFAs and protein quality could be related, as seen in a Canadian population [44], and thus affect muscle and whole-blood LC-PUFAs in parallel. The tissue most affected by malnutrition in the first months of life is muscle mass [45]. It could also be speculated that low MUAC is more related to long-term inflammation, which may be the cause of low PUFA status.

Markers of infection and inflammation were negatively associated with whole-blood PUFAs. This finding could be explained by an infection-induced supression of appetite and could be a direct effect of dietary intake, but may also be related to impaired absorption, poor nutrient utilization and/or increased nutrient catabolism caused by the inflammatory state [46, 47]. We speculate that the negative association of LA but positive association of DHA with diarrhea could be due to low absorption of fat during diarrhea resulting in low plasma triacylglycerols and thus, relatively higher levels of DHA from the blood cells. Fever during infection leads to an increase in energy requirement [48] and possibly an increased use of PUFAs as fuel. Infection-induced inflammation has been suggested to increase LC-PUFA catabolism, mainly of AA and DHA for the production of pro- and anti-inflammatory mediators [49] and is likely contributing to the reduced AA and DHA seen in the MAM children, specifically those with low MUAC. Finally, hemoglobin was found to positively correlate with whole-blood LC-PUFAs, mostly AA and DHA, which could possibly be due to their incorporation in phospholipids of erythrocyte membranes [14]. The negative association between hemoglobin and whole-blood LA could be caused by a preferential inorporation of LC-PUFAs in erythrocyte membranes at the expense of LA. The association with anemia and hemoglobin may therefore in part reflect changes in blood composition (erythrocyte number) and may therefore not be an ideal indicator of whole blood PUFA status.

The current study is the first to investigate whole-blood PUFA composition and correlates among a large sample of children with MAM. Also, this is the first study to show a relationship between low MUAC and PUFAs. The diversity of correlates such as infection, inflammation, diet, and anthropometry highlight the complexity of factors which may affect whole-blood PUFA composition in children with MAM, and these factors should be taken into account in studies investigating PUFAs among malnourished children. However, the study had some limitations, in particular the lack of a control group from the same study setting, which makes it difficult to differentiate between potential effects of diet and the overall low nutritional status. Differences in diet could confound differences in nutritional status even if we had been able to collect data from a well-nourished group of children in Burkina Faso. The diet information was based on a single qualitative 24-h recall which may have reduced accuracy and thus, reduced the association between diet and PUFAs. The observed associations were however meaningful. Furthermore, PUFA status was only based on a single non-fasting whole-blood sample and may not provide a measure of long-term status as is the case for example in adipose tissue samples [50], but whole-blood EPA and DHA correlates with and predicts erythrocyte EPA and DHA [51]. It may however, match the short-term collection of dietary information and also morbidity, which was based only on recall data from the previous 2 weeks and data from clinical examinations on the day of recruitment.

In conclusion, although PUFA deficiency based on the definition used here was not common, children with MAM in Burkina Faso have low whole-blood n-3 PUFA concentrations. Low n-3 PUFA levels are likely to be, to some extent, explained by a low intake of animal based foods, vegetable oils and fat sources of n-3 PUFA, or by other correlating factors such anemia, infection and inflammation. Children with low MUAC seem to have lower whole-blood PUFAs compared to children with MAM based on WHZ. This observation needs to be confirmed in future investigations among children with MAM.

## Abbreviations

AA: Arachidonic acid
AGP: α_1_-acid glycoprotein
ALA: α-linolenic acid
ANOVA: Analysis of variance
CI: Confidence interval
CRP: C-reactive protein
DHA: Docosahexaenoic acid
DPA: Docosapentaenoic acid
EPA: Eicosapentaenoic acid
FA%: Fatty acid percent
IQR: Interquartile range
LA: Linoleic acid
LC-PUFA: Long-chain polyunsaturated fatty acid
MAM: Moderate acute malnutrition
MUAC: Mid-upper-arm-circumference
MUFA: Monounsaturated fatty acid
PUFA: Polyunsaturated fatty acid
SAM: Severe acute malnutrition
SD: Standard deviation
SFA: Saturated fatty acid
WHO: World health organization
WHZ: Weight-for-height z-score
